# Supplementary material for: Advancing the Rose Rosette Virus Minireplicon and Encapsidation System by Incorporating GFP, Mutations, and the CMV 2b Silencing Suppressor
Source: Viruses. 2022 Apr 17;14(4):836. doi: 10.3390/v14040836 (PMC9031449; doi:10.3390/v14040836)
Supplement: Supplementary file 1 [file viruses-14-00836-s001.zip › Table S1.pdf]

Table S1. Primers used for cloning, RT-PCR and RT-qPCR

Name	Sequence	Nt Position	Function(s)
TAG	GGCCGTCATGGTGGCGAATA	N/A	PCR and qPCR
GFP209FT	GGCCGTCATGGTGGCGAATAAGTGCTTCAGCCGCTACCCC	209 - 228	Tagged-RT primer
GFP414RT	GGCCGTCATGGTGGCGAATATGTACTCCAGCTTGTGCCCC	414 - 433	Tagged-RT primer
GFP209F	AGTGCTTCAGCCGCTACCCC	209 - 228	PCR and qPCR
GFP414R	TGTACTCCAGCTTGTGCCCC	414 - 433	PCR and qPCR
R5GFPF-IF	AAGCTTGCACCAGTGATGGTGAGCAAGGGCGAGGA	Overlaps RNA5 and eGFP	Amplify eGFP, Cloning
R5GFPR-IF	AAATATATTGTAACTTTACTTGTACAGCTCGTCCATGCCGTGA	Overlaps RNA5 and eGFP	Amplify eGFP, Cloning
R5F	AGTTACAATATATTTGTTCCAATGA	176 - 200	Amplify RNA5, Cloning
R5R	CACTGGTGCAAGCTTTAAAAG	1605 - 1625	Amplify RNA5, Cloning
NbactinF	AAAGACCAGCTCATCCGTGGAGAA	128 - 151	PCR and qPCR
NbactinR	TGTGGTTTCATGAATGCCAGCAGC	249 - 272	PCR and qPCR
