# Supplementary figures and images for: Advancing the Rose Rosette Virus Minireplicon and Encapsidation System by Incorporating GFP, Mutations, and the CMV 2b Silencing Suppressor
Source: Viruses. 2022 Apr 17;14(4):836. doi: 10.3390/v14040836 (PMC9031449; doi:10.3390/v14040836)

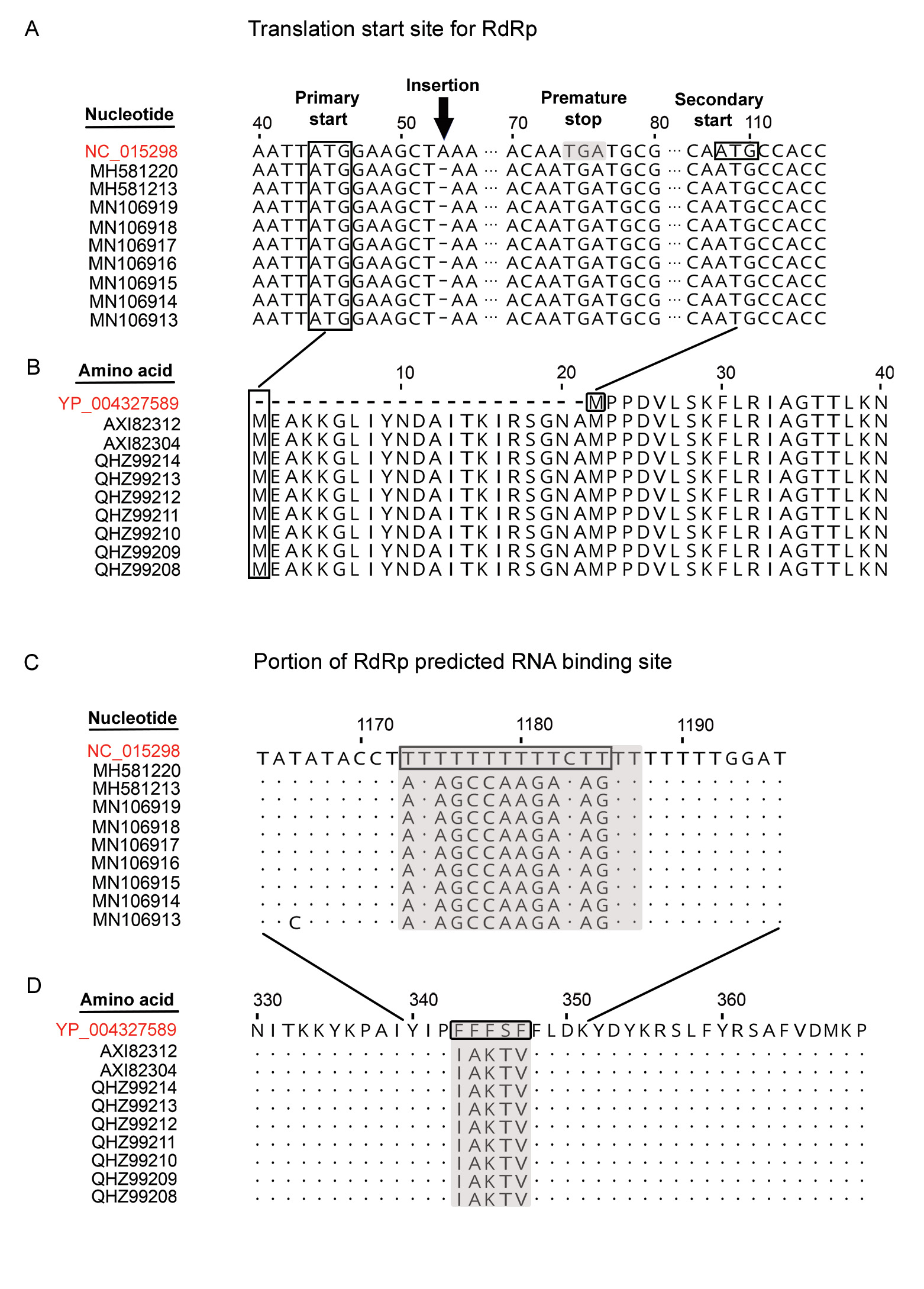

Supplement: Supplementary file 1 [file viruses-14-00836-s001.zip › Figure S1.jpg]

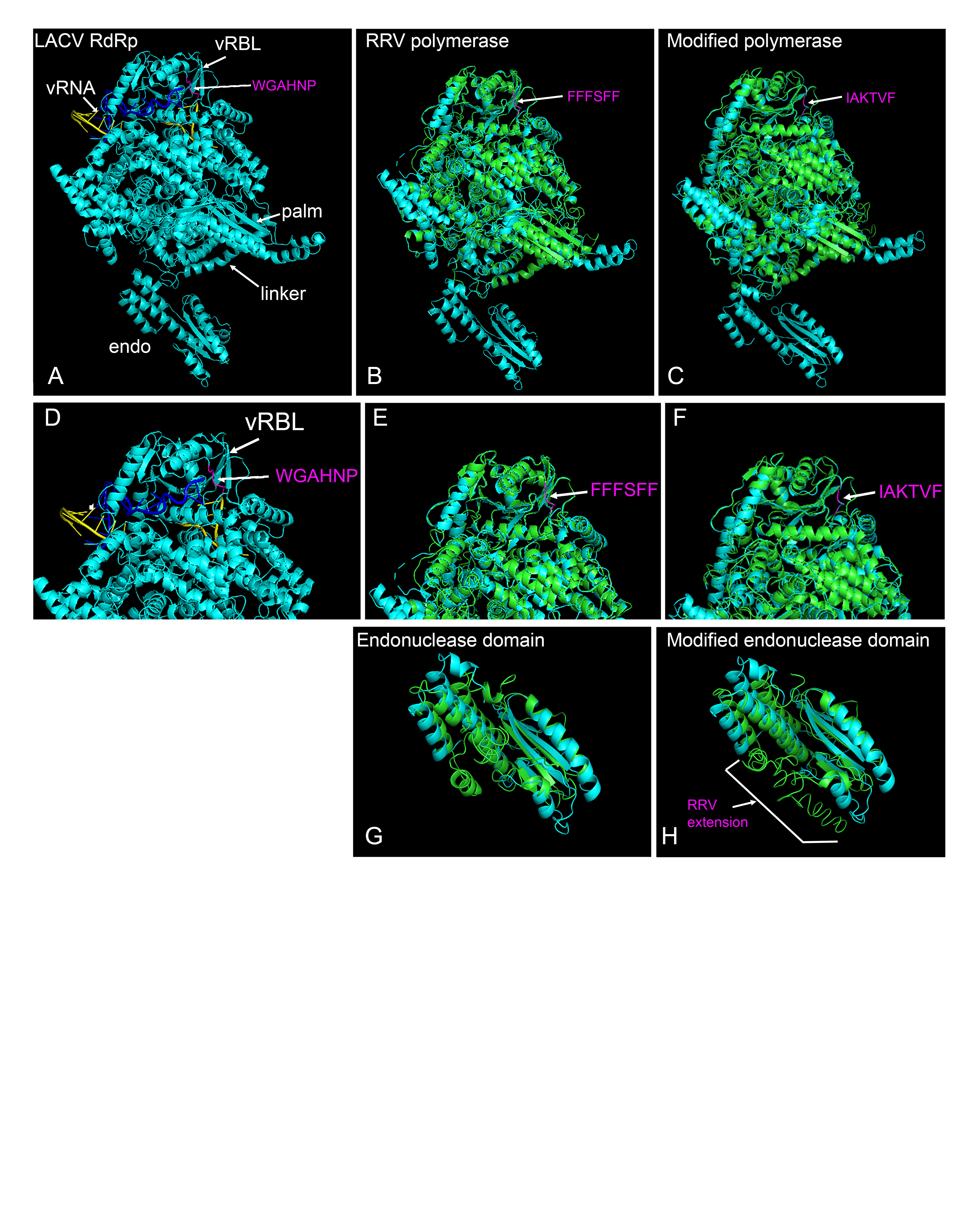

Supplement: Supplementary file 1 [file viruses-14-00836-s001.zip › Figure S2.jpg]

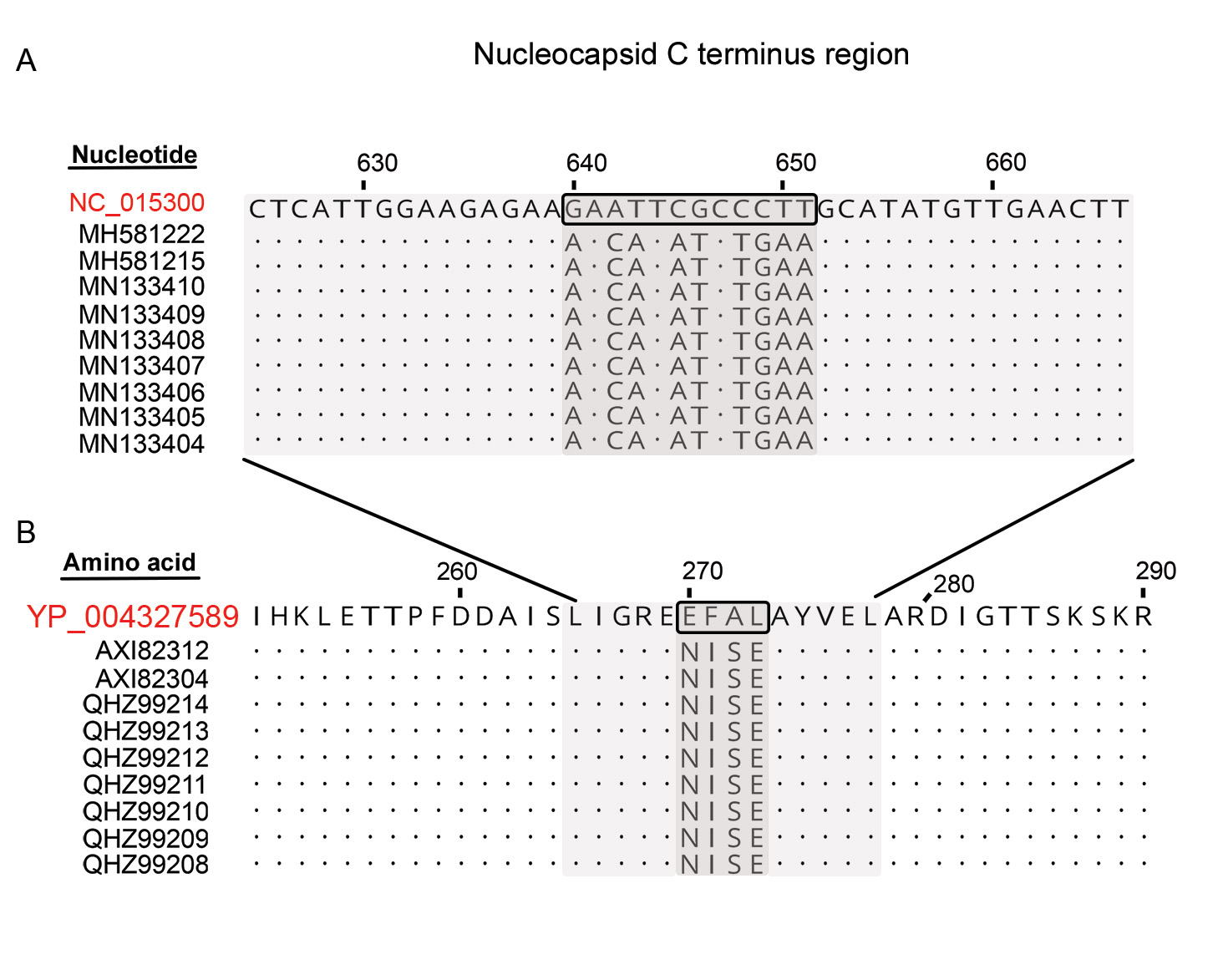

Supplement: Supplementary file 1 [file viruses-14-00836-s001.zip › Figure S3.jpg]

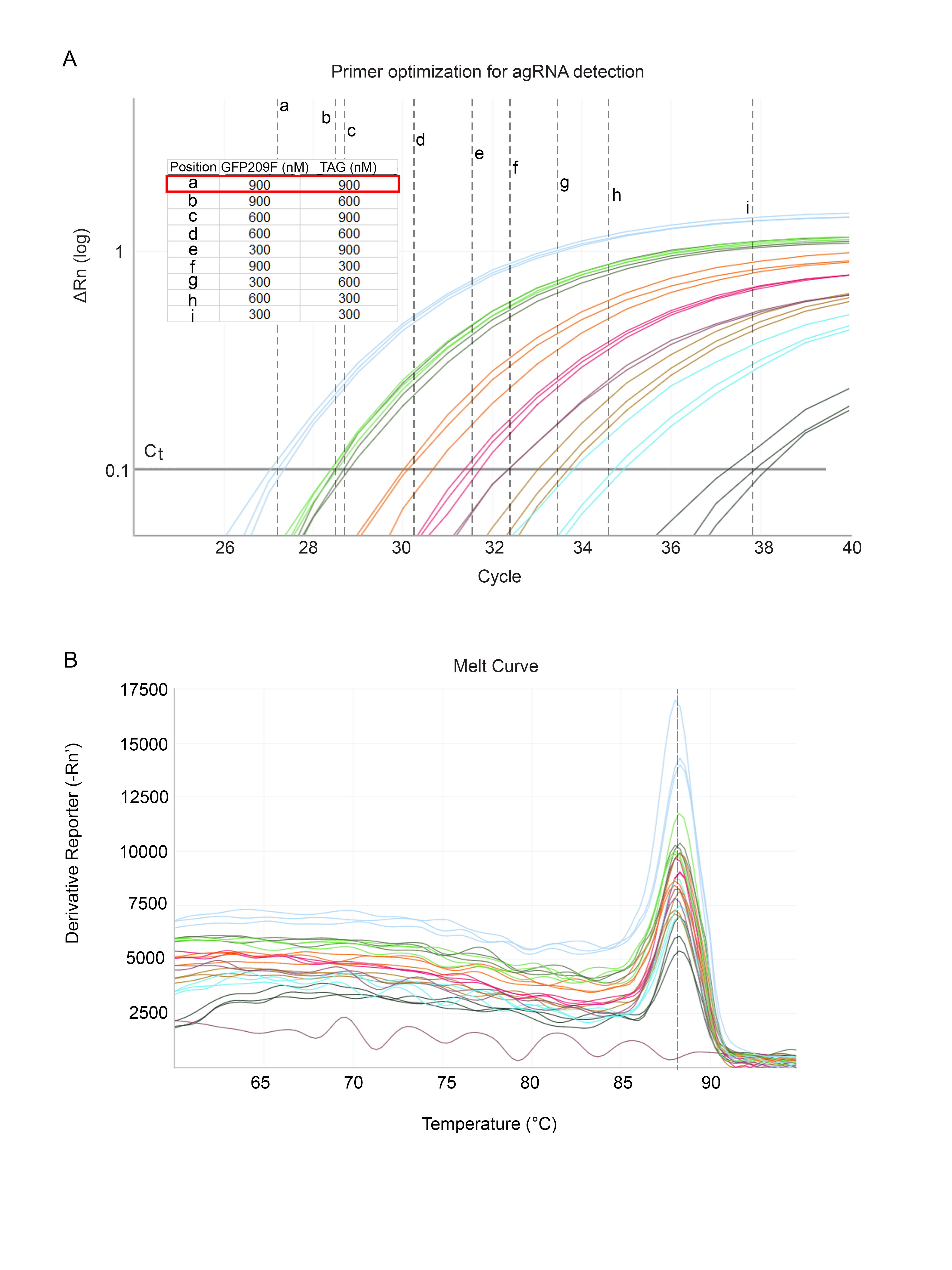

Supplement: Supplementary file 1 [file viruses-14-00836-s001.zip › Figure S4.jpg]

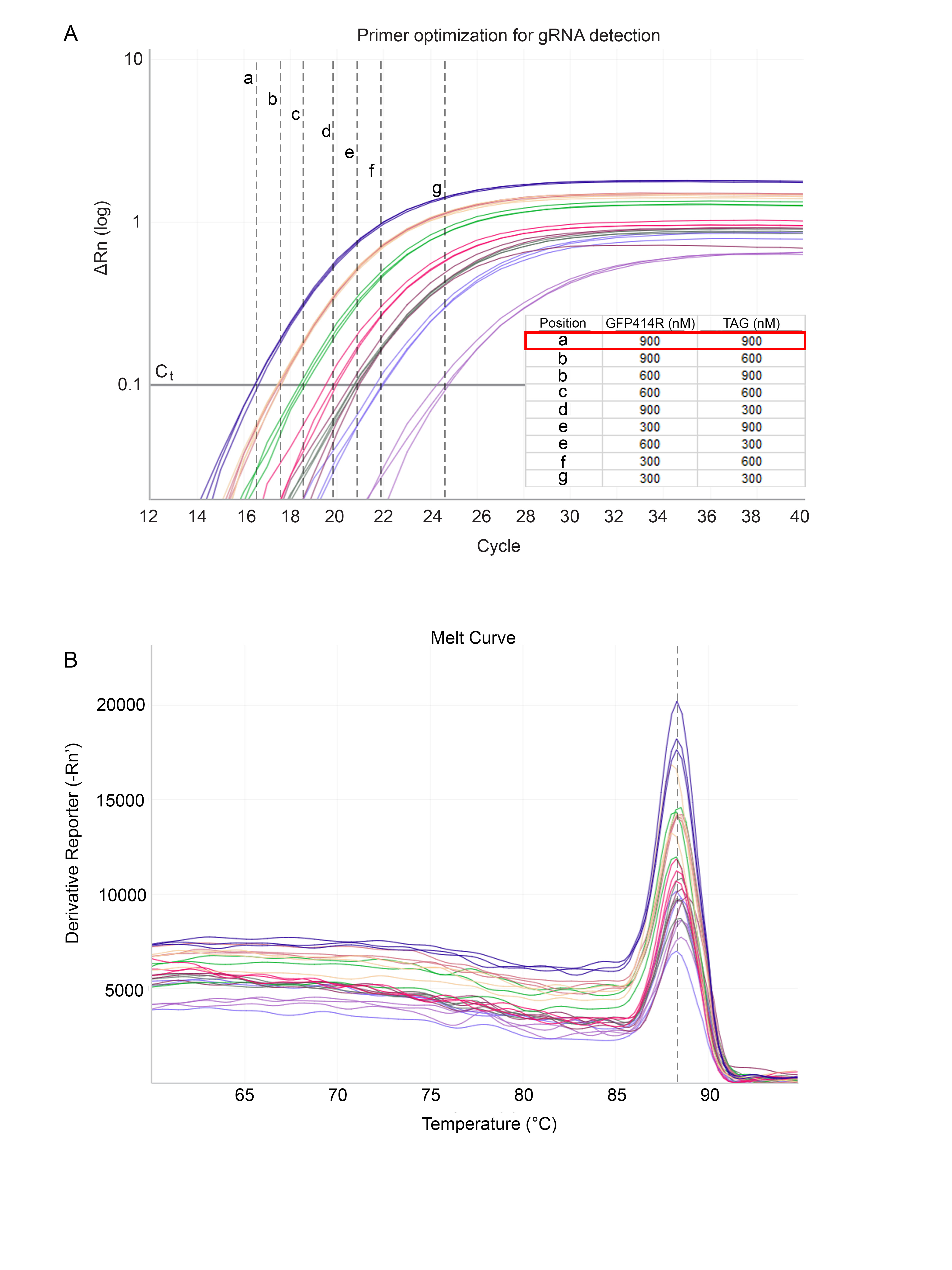

Supplement: Supplementary file 1 [file viruses-14-00836-s001.zip › Figure S5.jpg]
